# Immunophenotypic Analysis of Acute Megakaryoblastic Leukemia: A EuroFlow Study

**DOI:** 10.3390/cancers14061583

**Published:** 2022-03-21

**Authors:** Nienke Brouwer, Sergio Matarraz, Stefan Nierkens, Mattias Hofmans, Michaela Nováková, Elaine Sobral da Costa, Paula Fernandez, Anne E. Bras, Fabiana Vieira de Mello, Ester Mejstrikova, Jan Philippé, Georgiana Emilia Grigore, Carlos E. Pedreira, Jacques J. M. van Dongen, Alberto Orfao, Vincent H. J. van der Velden, on behalf of the EuroFlow Consortium

**Affiliations:** 1 Laboratory Medical Immunology, Department of Immunology, Erasmus MC, University Medical Center Rotterdam, Wytemaweg 80, 3015 CN Rotterdam, The Netherlands; nienke1.brouwer@wur.nl (N.B.); anne.bras@catharinaziekenhuis.nl (A.E.B.); 2 Translational and Clinical Research Program, Centro de Investigación del Cáncer (IBMCC; CSIC—University of Salamanca), Cytometry Service, NUCLEUS, Department of Medicine, University of Salamanca (USAL) and Institute of Biomedical Research of Salamanca (IBSAL) Paseo de la Universidad de Coimbra s/n, Campus Miguel de Unamuno, 37007 Salamanca, Spain; smats@usal.es (S.M.); orfao@usal.es (A.O.); 3 Biomedical Research Networking Centre Consortium of Oncology (CIBERONC), Instituto de Salud Carlos III, 28029 Madrid, Spain; 4 Princess Máxima Center for Pediatric Oncology, Heidelberglaan 25, 3584 CS Utrecht, The Netherlands; s.nierkens-2@prinsesmaximacentrum.nl; 5 Department of Laboratory Medicine, Ghent University Hospital, Corneel Heymanslaan 10, 9000 Ghent, Belgium; mattias.hofmans@uzgent.be; 6 CLIP-Department of Pediatric Hematology and Oncology, Second Medical Faculty, Charles University and University Hospital Motol, V Uvalu 84, 15006 Prague, Czech Republic; michaela_novakova@lfmotol.cuni.cz (M.N.); ester.mejstrikova@lfmotol.cuni.cz (E.M.); 7 Institute of Pediatrics (IPPMG), Federal University of Rio de Janeiro (UFRJ), R. Bruno Lobo, 50—Cidade Universitária, Rio de Janeiro—RJ 21941-912, Brazil; elainesc.ufrj@gmail.com (E.S.d.C.); fabivmello@gmail.com (F.V.d.M.); 8 Institute for Laboratory Medicine, Kantonsspital Aarau AG, Tellstrasse 25, 5001 Aarau, Switzerland; paula.fernandez@ksa.ch; 9 Department of Diagnostic Sciences, Ghent University, C. Heymanslaan 10, 9000 Ghent, Belgium; jan.philippe@ugent.be; 10 Cytognos SL, Carretera de Madrid Km. 0 Nave 9, Pol. La Serna, 37900 Salamanca, Spain; ggrigore@cytognos.com; 11 Systems and Computing Department, COPPE, Federal University of Rio de Janeiro (UFRJ), Rio de Janeiro-RJ 21941-594, Brazil; pedreira56@gmail.com; 12 Department of Immunology, Leiden University Medical Center (LUMC), Albinusdreef 2, 2333 ZA Leiden, The Netherlands; j.j.m.van_dongen@lumc.nl; 13 Centro de Investigación del Cancer, Department of Medicine, University of Salamanca, 37007 Salamanca, Spain

**Keywords:** EuroFlow, immunophenotyping, Down syndrome, transient abnormal myelopoiesis, AMKL

## Abstract

**Simple Summary:**

Acute megakaryoblastic leukemia (AMKL) is a rare and heterogeneous subtype of acute myeloid leukemia (AML). We show that such patients can be identified by flowcytometric immunophenotyping using the standardized EuroFlow panel. AMKL patients show a unique immunophenotypic profile, and among AMKL patients, various subgroups can be distinguished.

**Abstract:**

Acute megakaryoblastic leukemia (AMKL) is a rare and heterogeneous subtype of acute myeloid leukemia (AML). We evaluated the immunophenotypic profile of 72 AMKL and 114 non-AMKL AML patients using the EuroFlow AML panel. Univariate and multivariate/multidimensional analyses were performed to identify most relevant markers contributing to the diagnosis of AMKL. AMKL patients were subdivided into transient abnormal myelopoiesis (TAM), myeloid leukemia associated with Down syndrome (ML-DS), AML—not otherwise specified with megakaryocytic differentiation (NOS-AMKL), and AMKL—other patients (AML patients with other WHO classification but with flowcytometric features of megakaryocytic differentiation). Flowcytometric analysis showed good discrimination between AMKL and non-AMKL patients based on differential expression of, in particular, CD42a.CD61, CD41, CD42b, HLADR, CD15 and CD13. Combining CD42a.CD61 (positive) and CD13 (negative) resulted in a sensitivity of 71% and a specificity of 99%. Within AMKL patients, TAM and ML-DS patients showed higher frequencies of immature CD34+/CD117+ leukemic cells as compared to NOS-AMKL and AMKL-Other patients. In addition, ML-DS patients showed a significantly higher expression of CD33, CD11b, CD38 and CD7 as compared to the other three subgroups, allowing for good distinction of these patients. Overall, our data show that the EuroFlow AML panel allows for straightforward diagnosis of AMKL and that ML-DS is associated with a unique immunophenotypic profile.

## 1. Introduction

Acute myeloid leukemia (AML) is a heterogeneous malignancy characterized by clonal proliferation of myeloid precursor cells with differentiation arrest. The World Health Organization (WHO) classifies AML into different categories, including AML with recurrent genetic abnormalities, AML with myelodysplasia-related changes, AML not otherwise specified (NOS) and myeloid proliferation associated with Down syndrome [1,2,3,4]. The AML-NOS category is further classified into eight subgroups according to maturation, comparable to the French–American–British (FAB) classification system (AML subtypes M0-M7) [4].

AML-NOS with megakaryocytic differentiation (AML-M7; NOS-AMKL) is a rare but life-threatening subtype of AML affecting about 10% of pediatric AML patients and about 1% of adult AML patients [5]. These patients are characterized by the accumulation of ≥20% bone marrow leukemic cells with at least 50% showing overt megakaryocytic lineage commitment [4]. However, straightforward diagnosis of NOS-AMKL patients remains challenging due to their high biological and clinical heterogeneity [6,7,8]. Additionally, megakaryocytic differentiation of leukemic cells can also be observed in at least three other WHO subgroups. First, about 70–90% of patients with Down syndrome and myeloid leukemia (ML-DS) show megakaryocytic differentiation [1,9]. Second, about 10% of Down syndrome patients show transient abnormal myelopoiesis (TAM) in the first weeks after birth [10]. The circulating blasts in TAM virtually always show immunological features of megakaryocytic differentiation. The third subgroup concerns AML-NOS with megakaryocytic differentiation (NOS-AMKL) [4]. Finally, AMKL patients with a recurrent t(1;22) (RBM15-MKL1), often identified in early infancy, are separately classified as such [2,11]. The biological variation between these subgroups is significant, since each of them shows distinct genetic and molecular abnormalities [6,12]. Whereas TAM patients generally spontaneously clear the AMKL cells within a few weeks [13], the prognosis for other AMKL subtypes is rather unfavorable [5,14].

The development and application of flowcytometric immunophenotyping has significantly increased the accuracy of AMKL diagnosis. Blast identification in the bone marrow is performed by flowcytometric analysis, including staining of platelet-specific antigens (e.g., CD41, CD42b and CD61), stem/progenitor cell markers (e.g., CD34, CD33 and CD117), erythroid/megakaryocytic/monocytic markers (e.g., CD36) and T cell markers (e.g., CD7) [12,15,16,17,18,19]. In general, the immunophenotypic profiles of AMKL are well defined. Nevertheless, immunophenotypic differences between AMKL subgroups have only been studied to a limited extent, mainly due to the rarity of AMKL. Therefore, the aim of this study was to perform an immunophenotypic characterization of leukemic cells from 72 AMKL cases, as determined by the use of the standardized EuroFlow AML panel. The immunophenotypic profile of AMKL patients was compared with that of a control group of 114 non-AMKL AML patients. In addition, we searched for differential immunophenotypic profiles among subtypes of AMKL.

## 2. Materials and Methods

### 2.1. Patients

AMKL patients were selected for inclusion in this study if they fulfilled one of the following criteria: (1) WHO diagnosis of ML-DS, TAM, NOS-AMKL or t(1;22)-positive AML; (2) morphological FAB classification M7 or flowcytometric characteristics of megakaryocytic differentiation. A control group of non-AMKL AML patients was selected based on the availability of complete flowcytometric data from AML tube 1 to 7 (EuroFlow AML panel). All data were collected in seven centers between 2010 and 2021; the institutional review board of each participating center approved this study.

### 2.2. Immunophenotyping

Flowcytometric immunophenotyping was performed using the EuroFlow AML panel according to EuroFlow protocols and instrument settings [19,20,21]. Briefly, bone marrow (BM) or peripheral blood (PB) samples were stained with the appropriate cocktail of antibodies (30 min at room temperature (RT)), after which 2 mL FACS lysing solution (BD Biosciences, Erembodegem, Belgium) was added (10 min, RT). After centrifugation (540× *g*, 5 min), the cell pellet was washed with 2 mL phosphate-buffered saline (PBS) containing serum 0.2% bovine albumin (BSA), and cells were resuspended in 100 µL FACS flow buffer (BD Biosciences), with the exception of tube 4. TdT antibody (10 µL; clone HT-6, Agilent Technologies, Glostrup, Denmark) was added to the washed cells of tube 4, followed by incubation for 15 min at RT. After washing with PBS/BSA, cells were resuspended in FACS flow buffer. All samples were acquired on FACS canto II or FACS lyric flow cytometers set up according to the EuroFlow protocols [21,22]. In the EuroFlow panel (tube 6), CD42a and CD61 are combined in the same fluorescence channel and reported as CD42a.CD61.

### 2.3. Data Collection and Evaluation

For each AML case, we requested that the participating center upload the anonymized flow cytometry standard (FCS) files (raw data from the EuroFlow AML panel) to the secured EuroFlow server. In addition, the participating laboratories performed a basic analysis on merged FCS files (removal of debris/doublets and identification of lymphocytes) using only the backbone markers (allowing a uniform analysis across tubes) (Infinicyt software, Cytognos, Salamanca, Spain). The resulting analyzed (CYT) file was also uploaded to the secured server. In addition, a spreadsheet with patient annotations (e.g., age, gender, WHO classification and flow cytometer used) was requested. Finally, each CYT file was checked by an independent reviewer to make sure all cells were gated correctly.

### 2.4. Quality Assessment (QA) Procedure

Our QA strategy was based on lymphocytes because lymphocytes (1) were present in virtually all samples, (2) could easily be distinguished from myeloid cells (based on the backbone markers) and (3) were assumed to be unaffected in AML patients (as they originate from the lymphoid lineage). QA was performed as described by Bras et al. (Bras et al., submitted). Briefly, a reference file containing a fixed number of lymphocytes extracted from all tubes/cases was created, and data were visualized using principal component (PC) analysis (PCA) in PC1 vs. PC2 plots. Subsequently, the two-standard-deviation (SD) curves were represented within the PCA plots (one for the backbone markers and six separate ones for the tube-specific antibodies). Then, for each patient/tube, the lymphocyte population was compared with these reference 2SD curves. Patients with more than 20% of their lymphocyte population falling outside the 2SD curves were checked for errors and, if necessary, excluded from the dataset.

### 2.5. Data Analysis and Statistics

Data analysis was performed using Infinicyt (Cytognos, version 2.0), R Studio (version 1.4.1717), GraphPad Prism (version 5.04), Microsoft Excel (2016), MedCalc (https://www.medcalc.org/; accessed on 9 February 2022) and SPSS Statistics (version 25). For each patient, the mean fluorescence intensity (MFI) of leukemic cells was obtained for each marker. Overall, four main leukemic cell maturation stages were defined based on CD34 and CD117 expression. Additionally, differentiation of CD34+/CD117+ cells towards the neutrophil granulocytic lineage was defined by CD15 expression, towards B cells by CD19 expression, towards the monocytic lineage by CD64 expression and towards the erythroid/megakaryocytic lineages by CD36 expression in the absence of monocytic markers (CD64). For all markers, MFI values were defined, and a marker was considered to be positive if the MFI value was >1000, as previously reported [23,24,25]. Data were analyzed using PCA (APS tool in Infinicyt software), the CA tool in Infinicyt software (based on multiclass linear discrimination analysis) [26] and neighborhood automatic population separator (NAPS in Infinicyt software). Based on neighborhood components analysis (NCA) [27,28], NAPS is a powerful and solid sustained algorithm from the mathematical point of view. It aims to learn from the available data a distance metric that maximizes the performance of nearest-neighbor (NN) classification. NCA approaches the ‘neighborhood’ concept in a stochastic manner in the sense that each data point (corresponding to an individual case) selects another data point as its neighbor with a given probability. NCA calculates a transformation matrix from the marker space so that NN performance is optimized in the transformed space. Accordingly, the probability that data points (cases) are correctly classified can be computed, whereas the expected number of correctly classified cases can be maximized. The NAPS graphics represent the relative position of the points (like in clustering figures, such as t-SNE), and therefore, they do not depict any scale.

To examine statistical differences in the distribution of continuous variables, the non-parametric Kruskal–Wallis (for >2 groups) and/or Mann–Whitney U tests (for 2 groups) were used. *p*-values < 0.05 were considered statistically significant.

## 3. Results

### 3.1. Patient Characteristics

A total of 72 AMKL patients were included in this study, and all passed the QA. This group included 24 TAM, 16 ML-DS, 22 NOS-AMKL and 10 AMKL-Other patients (details in Table 1). The AMKL-Other patients, morphologically and/or immunophenotypically considered AML with megakaryoblastic maturation, included one AML patient with inv(16), one AML patient with NPM1-mutations, six AML patients with myelodysplasia-related changes and two AML patients without further classification. As expected, significant age differences were present between the four groups of patients, with only infants in the TAM group and a vast majority of children in the ML-DS group and the NOS-AMKL group (Figure S1A). In contrast, the AMKL-Other group mainly contained adult patients (median age >60 years old). WBC counts were also significantly different between the four groups, with the highest levels in the TAM and NOS-AMKL patients (Figure S1B). Most tested TAM cases showed GATA1 mutations (17/19; 89%). Other molecular data were only limitedly available: t(1;22) translocations (resulting in *RBM15-MKL1* fusion) were detected in only three patients, and given the low number, they were not analyzed separately but included in the NOS-AMKL group.

For the non-AMKL group, a total of 114 AML patients passed QA and were included (details in Table 1). The median age was 14 years old (due to composition of participating laboratories, with half of the centers being pediatric oncology centers). WBC counts were similar to TAM and NOS-AMKL counts but significantly higher than in ML-DS and AMKL-Other (Figure S1B).

### 3.2. Immunophenotypic Profile of AMKL versus Non-AMKL Patients: Univariate Analysis

Immunophenotypic profiles of the 72 AMKL patients were compared with those of the control group of 114 non-AMKL patients. A heatmap visualizing the MFI values for all evaluated markers of AMKL and non-AMKL patients is shown in Figure S2. In the majority of AMKL patients, the blast population showed relatively high expression of the megakaryocytic markers CD42a.CD61, CD41 and/or CD42b as compared to the non-AMKL population. Additionally, CD36, CD7, CD33, CD38 and CD71 were also frequently expressed in AMKL; however, expression of these markers was also frequently observed in non-AMKL patients. HLADR and CD13 expression was often found to be low or absent in AMKL, whereas non-AMKL showed HLADR and/or CD13 expression in the vast majority of cases (Supplementary Figure S2). The number of patients positive for these markers in the AMKL and non-AMKL groups, as well as odds ratios, are shown in Table 2. The vast majority of AMKL patients (>95%) showed positivity for CD41, CD42b and/or CD42a.CD61, whereas these markers were much less frequently detected in non-AMKL patients (<20%). AMKL patients also showed a significantly higher frequency of CD36 (92% vs. 60%), CD71 (90% vs. 57%) and CD7 expression (70% vs. 32%). Conversely, expression of other markers was significantly less frequent in AMKL versus non-AMKL patients, including HLADR, CD13, CD123 and expression of the lineage commitment antigens CD11b, CD15, CD64, CD14, CD300e and CD203c (Table 2). Univariate logistic regression analysis demonstrated that the highest predictive value for AMKL diagnosis was shown by the expression of CD42b (odds ratio (OR): 119), followed by CD42a.CD61 (OR: 103), CD41 (OR: 15), CD36 (OR: 7.3), CD71 (OR: 7.0) and CD7 (OR: 5.2) (Table 2).

### 3.3. Expression of Megakaryocytic Markers

The above data show that AMKL patients have a specific immunophenotypic profile, characterized overall by expression of megakaryocyte lineage-associated markers but generally lacking CD13 and HLADR expression. Since expression of megakaryocytic markers is a prerequisite for classifying an AML as an AMKL [4], we investigated the diagnostic contribution of EuroFlow panel tubes 6 and 7. As shown in Supplementary Figure S3, CD41a.CD61 was most frequently expressed in AMKL patients (59/65; 91%), followed by CD42b (52/65; 80%) and CD41 46/65 (71%). All three megakaryocytic markers were present in 39/65 (60%) of AMKL cases. Three cases had MFI levels below 1000 for all three megakaryocytic markers; one of these concerned an ML-DS patient, and the other two were classified as NOS-AMKL (Supplementary Figure S4). Among the non-AMKL patients, 22 expressed at least one megakaryocytic marker; CD42a.CD61 was positive in 11/114 (10%), CD41 in 15/114 (13%) and CD42b in 4/114 (4%) cases. Most cases (14/22) expressed only one marker, generally weakly, and coexpression of all three markers was never observed. Monocytic differentiation was present in 16/22 cases. Details of the non-AMKL patients expressing at least one megakaryocytic marker are provided in Table S1. Overall, these data show that CD42a.CD61 was the most sensitive marker for AMKL detection (91%), whereas CD42b was the most specific marker (96%).

### 3.4. Immunophenotypic Profile of AMKL versus Non-AMKL: Multivariate Analysis

**Figure 1:**
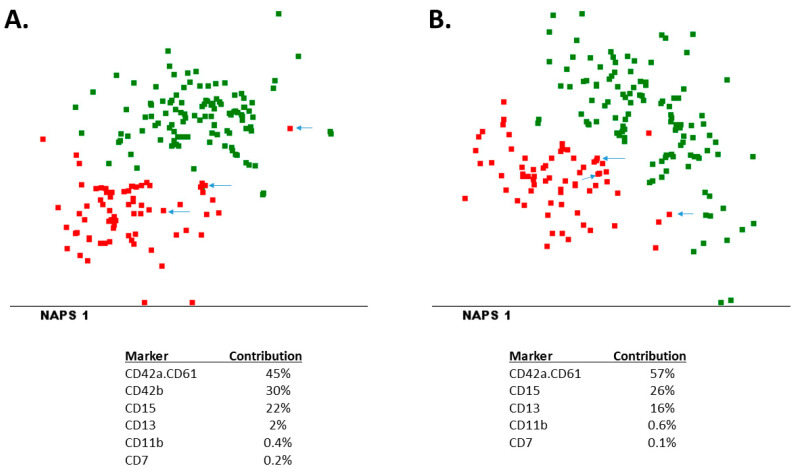
Multivariate analysis of non-AMKL (green dots) and AMKL patients (red dots) using the MFI values of all markers present in EuroFlow AML tubes 1–7 (**A**) or tubes 1–6 (**B**). Pattern classification was performed using NAPS, and the markers contributing to the pattern classification are shown in the bottom part of the figure. The three AMKL patients not expressing CD42a.CD61, CD41 or CD42b (MFI < 1000) are indicated by arrows.

We used the MFI values of all markers as input for multidimensional analyses (combining the information of all 32 markers). As shown in Figure 1A, AMKL patients could clearly be distinguished from non-AMKL patients, particularly based on CD42a.CD61, CD42b, CD15, CD13, CD11b and CD7. One of the three AMKL patients without expression of megakaryocytic markers (ML-DS 3) was located in the same area of the plot as the non-AMKL patients, whereas the other two patients (NOS-AMKL 15 and NOS-AMKL 16) were located on the border between the two patient groups. These data show that most AMKL patients can be distinguished from non-AMKL patients using EuroFlow AML panel tubes 1 to 7. Similar analyses were performed using the data from tubes 1 to 6 only (Figure 1B), obtaining highly comparable plots and contributing markers, which confirms that using tubes 1 to 6 is sufficient for AMKL diagnostics and that tube 7 is mainly for confirming differentiation of blast cells to the megakaryoblastic lineage.

Linear regression analysis showed a significant contribution of CD13, HLADR, CD34 and CD42a.CD61 in distinguishing AMKL and non-AMKL patients. The presence of a CD13-/CD34+/HLADR-/CD42a.CD61+ immunophenotype resulted in a sensitivity of 40% and 100% specificity. Without HLADR, the sensitivity increased to 54%, with a similar 100% specificity. Including only CD42a.CD61 and CD13 resulted in a sensitivity of 72% and a specificity of 99%.

### 3.5. Maturation-Stage-Related Immunophenotypic Profiles

**Figure 2:**
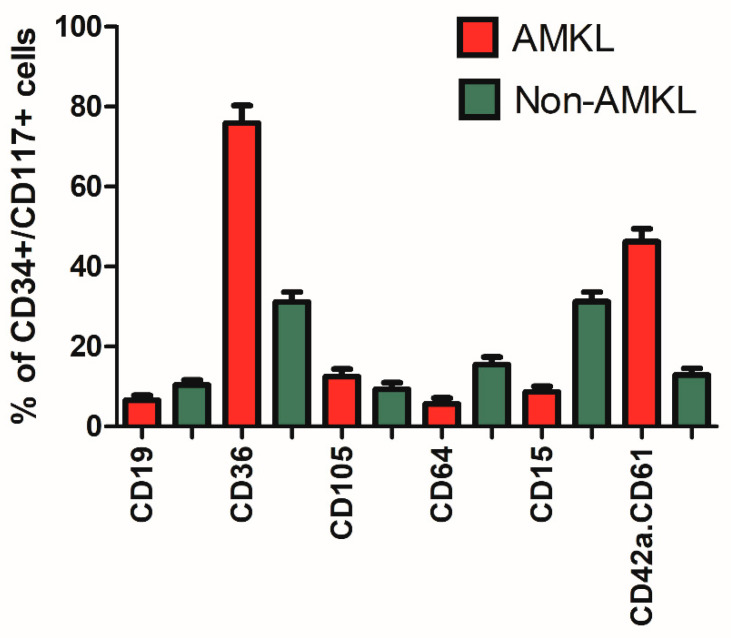
Differentiation of CD34+/CD117+ AML cells in AMKL (red bars) and non-AMKL patients (green bars). Percentage of positive cells, defined as cells with an MFI > 1000 (mean ± SEM). Differentiation towards B-cell (CD19+), megakaryocytic/erythroid (CD36+/CD64−), erythroid (CD105+), monocytic (CD64+), granulocytic (CD15+) and megakaryocytic (CD42a.CD61+) lineage is shown.

Since gating/identification of AML cells can be challenging due to the heterogeneity of the blast cell population and their similarities with normal myeloid cells, we evaluated whether focusing on the most immature CD34+/CD117+ cells might facilitate the analysis. After gating on CD34+/CD117+ cells (using MFI values of 1000 as cutoff), differentiation towards the B-cell (CD19+), megakaryocytic/erythroid (CD36+/CD64−), erythroid (CD105+), monocytic (CD64+), neutrophilic (CD15+) and megakaryocytic (CD42a.CD61+) lineage was defined in both the AMKL and non-AMKL patients. As shown in Figure 2, leukemic cell differentiation of AMKL patients was expectedly skewed towards the megakaryocytic/erythroid lineage, whereas immunophenotypic commitment toward other cell lineages was significantly lower as compared to non-AMKL patients. Multivariate analysis performed on CD34+/CD117+ cells from AMKL and non-AMKL patients showed good discrimination of the two groups, both using tubes 1–6 and tubes 1–7 with major contribution of CD42a.CD61 and CD36 in both comparisons (Supplementary Figure S5). However, multidimensional separation of AMKL versus non-AMKL patients was not significantly improved when focusing on CD34+/CD117+ leukemic cells.

**Figure 3:**
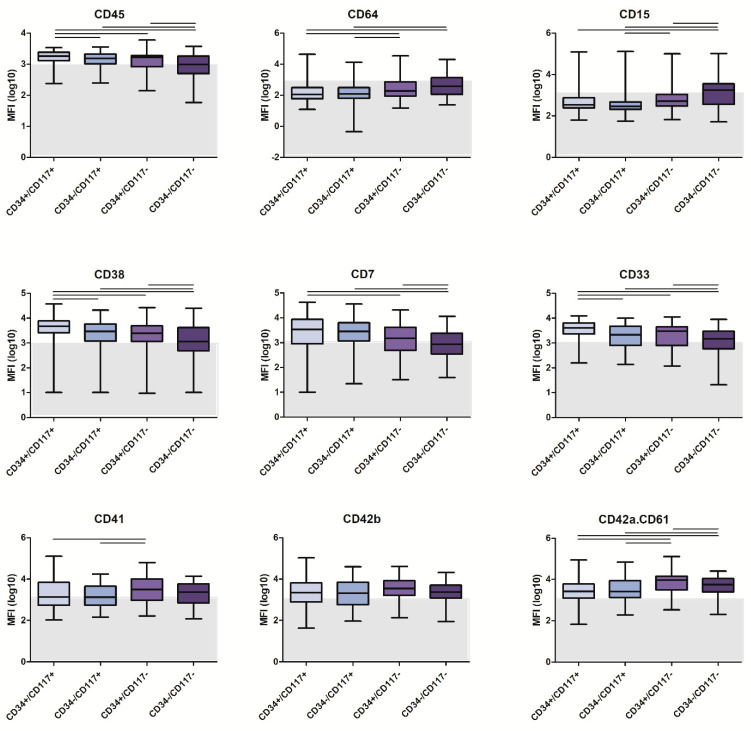
Immunophenotypic profile of CD34+/CD117+, CD34−/CD117+, CD34+/CD117− or CD34−/CD117− subsets of AMKL cells. Expression of markers is depicted as log10 transformed MFI data. Statistical analysis was performed by the Kruskal–Wallis test, followed by the Mann–Whitney test if *p* < 0.05. The horizontal lines between populations represent statistically significant differences (*p* < 0.05). The grey zone indicates MFI levels < 1000; markers with such MFI values were considered to be negative.

Given the observed heterogeneity in expression of CD34 and CD117 in AMKL patients, we further evaluated whether there were immunophenotypic differences between various maturation stages within the AMKL patients. Thus, for all AMKL patients, AML cells were divided into CD34+/CD117+, CD34−/CD117+, CD34+/CD117− and CD34−/CD117− subsets (based on the backbone markers present in all tubes). As shown in Figure 3 and Supplementary Figure S6, several markers, including maturation markers, such as CD11b, CD14 and CD64, were more strongly expressed in the CD34−/CD117− subset, which is in line with their presumed higher degree of differentiation. In contrast, CD13, CD33, CD7 and CD38 expression was higher in the CD34+/CD117+ subset. The megakaryocytic markers (CD41, CD42b, CD42a.CD61 and CD36) showed rather similar expression between the four maturation subsets, although their expression was generally highest in the CD117−negative subsets. In turn, CD123 was most strongly expressed in the CD34+/CD117− subset (Supplementary Figure S6).

Multidimensional analysis of the four maturation subsets showed that, in particular, the CD34+/CD117− subset could be relatively well distinguished from the other three subsets, with CD15, CD123, CD11b and CD36 as the most contributing markers (Supplementary Figure S7). The CD34−/CD117+ subset could be separated from the CD34−/CD117− subset mainly by CD15, whereas distinguishing other subsets (e.g., CD34+/CD117+ versus CD34−/CD117−) was not possible. Overall, these data show that the different maturation subsets have some differences in immunophenotypic profiles but that most markers are similarly expressed.

### 3.6. Immunophenotypic Variability within AMKL

**Figure 4:**
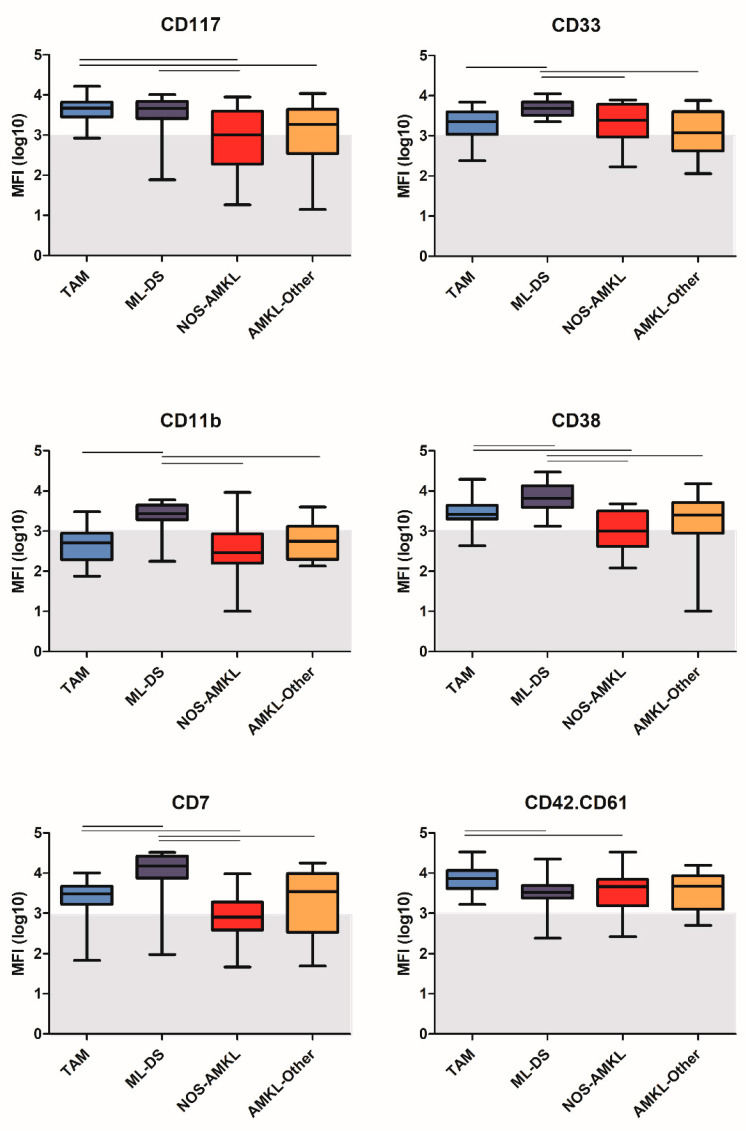
Univariate analysis of marker expression between the four AMKL subgroups. Data represent the MFI values after log10 transformation. The lines the on top of the figures represent statistically significant differences (*p* < 0.05) between the two groups. The grey zone indicates MFI levels < 1000; markers with such MFI values were considered to be negative.

**Figure 5:**
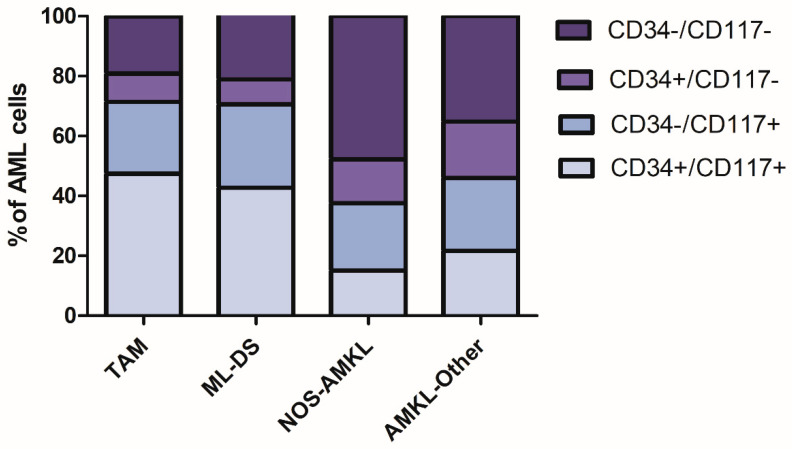
Distribution of AMKL cells over the various maturation stages, as defined by CD34 and CD117 expression. The percentage of CD34+/CD117+ leukemic cells was significantly higher in TAM and ML-DS patients as compared to NOS-AMKL and AMKL-Other patients (*p* < 0.05 by Mann–Whitney test); in contrast, the percentage of CD34−/CD117− leukemic cells was higher in the NOS-AMKL patients (*p* < 0.05) and AMKL-Other patients (not significant).

We next evaluated the immunophenotypic profiles of the four AMKL subgroups included in our study: TAM, ML-DS, NOS-AMKL and AMKL-Other. Leukemic cells from all patient groups were typically positive for CD34, CD117, CD45 and CD36. Univariate analysis revealed that ML-DS patients showed a significantly higher expression of CD45, CD33, CD35, CD38, CD11b and CD7 as compared to the other three groups (Figure 4 and data not shown). In turn, TAM and ML-DS patients showed significantly higher expression levels of CD34 and CD117 on leukemic cells as compared to NOS-AMKL patients. Further analysis showed a higher frequency of CD34+/CD117+ immature cells in TAM and ML-DS patients as compared to NOS-AMKL and AMKL-Other patients (Figure 5). The vast majority of blasts in all four subgroups showed expression of CD41, CD42 and CD42a.CD61; however, expression of CD42b and CD42a.CD61 (Figure 4) was significantly higher in TAM as compared to ML-DS and NOS-AMKL patients, whereas CD41 expression was comparable across the four subgroups.

**Figure 6:**
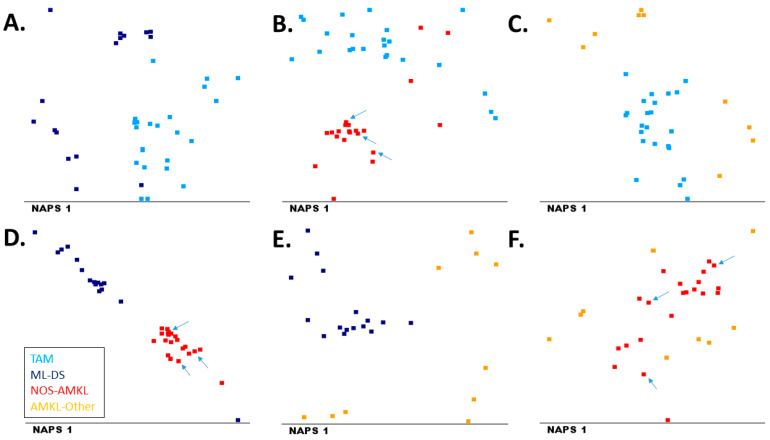
Multivariate analysis of marker expression between the four AMKL subgroups. (**A**) TAM versus ML-DS (contributing markers: CD7 (47%), CD11b (31%) and CD13 (21%)); (**B**) TAM versus NOS-AMKL (contributing markers: CD117 (28%), CD4 (21%) and CD42b (19%)); (**C**) TAM versus AMKL-Other (contributing markers: CD117 (61%), CD13 (36%) and CD19 (3%)); (**D**) ML-DS versus NOS-AMKL (contributing markers: CD7 (68%), CD117 (27%) and CD11b (3%)); (**E**) ML-DS versus AMKL-Other (contributing markers: CD33 (51%), CD203c (28%) and CD38 (21%)); (**F**) NOS-AMKL versus AMKL-Other (contributing markers: CD7 (97%), CD38 (2%) nad CD117 (0.4%)). The arrows indicate NOS-AMKL patients with t(1;22).

Finally, we evaluated whether the four groups could be discriminated from one another in multivariate analysis. Multidimensional analysis was performed using the mean expression values of all markers as input (combing the information of all 32 markers). As shown in Figure 6, TAM, ML-DS and NOS-AMKL patients were generally well separated, whereas the AMKL-Other patients were more variable and showed less separation from the other three groups. In line with the univariate analysis, markers distinguishing the AMKL subgroups included CD7, CD11b, CD33 and CD117. The three NOS-AMKL patients with t(1;22) showed a comparable immunophenotype to that of the other NOS-AMKL patients (Figure 6).

## 4. Discussion

AMKL is a rare and heterogeneous disease. In this paper, we showed that more than 95% of AMKL cases show a specific immunophenotype, with expression of megakaryocytic markers in most cases, and generally lack CD13 and HLADR. Most AMKL patients show heterogeneous expression of CD34 and CD117, but these different subsets show rather comparable immunophenotypes. However, TAM and ML-DS patients overall have higher percentages of CD34+/CD117+ immature cells as compared to NOS-AMKL and AMKL-Other patients (46% vs. 19%).

To make a diagnosis of AMKL, the leukemic cells should express megakaryocytic markers [4]. Interestingly, three cases in our AMKL series did not show such expression and clustered together with or near non-AMKL patients in the multivariate analysis. One such case was an ML-DS patient; the minority (about 5–10%) of such patients are known to not show megakaryoblastic differentiation. Despite the lack of megakaryocytic markers in immunophenotyping, this case clustered with the remaining ML-DS cases in the multivariate analysis with other AMKL subgroups, indicating that besides the differences in megakaryocytic markers, other markers were more comparably expressed. Morphologically, the leukemic cells were megakaryoblasts. The same was true for the other two patients, who were negative for megakaryocytic markers and classified as NOS-AMKL. Although their immunophenotype may have some characteristics of megakaryoblastic lineage, formally, these cases should not have been diagnosed as NOS-AMKL. The 10 AMKL-Other cases in our study had immunophenotypic features of megakaryocytic differentiation but were WHO-classified differently, mostly as AML with myelodysplasia-related changes. Since 149/306 (49%) of MDS patients show abnormal megakaryocytic cells [29], this finding is not unexpected. On the other hand, analysis of non-AMKL patients showed expression of at least one megakaryocytic marker in 22/114 (19%) of the cases and expression of CD42a.CD61 (present in tube 6) in 11/114 (10%) of cases. Most cases (14/114; 12%) expressed only one megakaryocytic marker, and expression was generally weak. Furthermore, most cases (16/22; 73%) appeared to exhibit monocytic differentiation. Non-specific binding of platelets to monocytic AML cells may have resulted in seemingly positive expression of megakaryocytic markers in these cases, but this did not affect their classification. Clearly, next to megakaryocytic markers, other markers should also be considered when immunophenotypically classifying patients. Using EuroFlow AML panel tubes 1 to 6, appropriate diagnosis of AMKL patients is possible, and tube 7 is particularly designed for confirming the AMKL diagnosis. Indeed, our data show that CD42a.CD61 was most sensitive, whereas CD42b was most specific. Based on our data, a combination of the backbone markers CD34, CD117, HLADR and CD45 supplemented with CD13, CD42a.CD61, CD36 and CD42b may allow for flowcytometric confirmation of AMKL diagnosis in patients highly suspected for AMKL based on morphological and/or clinical criteria.

AMKL patients often showed heterogeneous expression of CD34 and CD117, which may hamper easy gating of the leukemic cells. When focusing on the presumed most immature CD34+/CD117+ cells, we found that these cells were more frequent in ML-DS and TAM patients (47% and 44%, respectively, versus 15% in NOS-AMKL and 22% in AMKL-Other), which is in line with the observed increase in CD34^+^ immature megakaryoblasts by the GATA1 mutation [30]. Immunophenotypically, the CD34+/CD117+ cells in the AMKL patients clearly showed megakaryocytic differentiation, with frequent expression of CD36 (76% of cases) and/or CD42a.CD61 (46% of cases) but lacking markers indicative of other lineages (such as CD19, CD15 and CD64). In multidimensional analysis, AMKL patients could be generally well separated from non-AMKL patients, both for the total leukemic population as well as for the CD34+/CD117+ cells. This confirms the results of an older study, which showed that the CD34+ cells in AMKL are different from those in AML M0-M6 cases [15]. Comparing CD34+/CD117+ cells of AMKL patients with CD34−/CD117+, CD34+/CD117− or CD34−/CD117− cells showed some differences, e.g., higher expression of CD15 and CD64 in the CD34−/CD117− cells, indicating some maturational changes. However, it should be noted that such differences might also at least partially be explained by contamination of the leukemic cell gate by mature neutrophils and/or monocytes. The observation that the megakaryocytic markers were comparably expressed by the four different maturation stages indicates that these markers were already acquired early during megakaryocytic ontogeny.

AMKL patients consisted of four different groups, and immunophenotypic differences between these groups could be observed. ML-DS patients have been reported to exhibit a specific immunophenotypic profile (CD7+/CD36+/CD117+/CD71+/CD4low/CD42b+) [17], which was confirmed in this study. Although TAM and ML-DS are related to one another, ML-DS and TAM patients showed differences in marker expression. For example, expression of CD33, CD7, CD38 and CD11b were higher in ML-DS patients, extending and confirming previous studies [12,18]. Another study showed that deficiency of CD11a is a marker for TAM and AMKL [31]; however, this CD11a is not included in the EuroFlow protocol. CD36 was highly expressed in both TAM and ML-DS patients; expression of this marker has been associated with high drug sensitivity [32]. In multidimensional analyses, TAM and ML-DS cases were generally separated from NOS-AMKL and AMKL-Other patients, whereas the latter two groups could not be separated well and also seemed more heterogeneous.

In our study, we used mean fluorescence intensities (MFI) for evaluation of marker expression in multidimensional analysis. One of the limitations of this parameter could be the susceptibility for outliers in comparison to median values. However, given the heterogeneous expression of several markers, with some markers only expressed by subpopulation(s), the mean value seemed to be more appropriate than a median MFI, which would not take such expression into account, or only to a limited extent.

## 5. Conclusions

In conclusion, AMKL generally can be reliably diagnosed using the EuroFlow AML panel (tubes 1–6). Besides the prominent contribution of the megakaryocytic markers, other markers (such as CD36, HLADR and CD13) may support the diagnosis. Among AMKL patients, TAM and ML-DS groups show a specific immunophenotypic profile. A future challenge is to obtain more insights into unique immunophenotypes related to underlying molecular abnormalities of AMKL subgroups in order to improve AMKL identification in future diagnostics. The use of standardized diagnostic protocols, such as the EuroFlow panel, will facilitate the collection of a sufficient number of patients with comparable flowcytometric data.

## Figures and Tables

**Table 1:** Patient characteristics.

			AMKL			Non-AMKL
	TAM	ML-DS	NOS-AMKL	AMKL-Other	all	
*n*	24	16	22	10	72	114
Age in years (median, range)	0 (0–0)	1 (0–4)	1 (0–89)	62 (2–86)	1 (0–89)	14 (0–93)
Gender (M/F)	12/12	8/8	13/9	6/4	39/33	60/54
WBC × 10^9^/L (median, range)	49 (7–179)	6 (2–35)	21 (3–94)	4 (2–16)	14 (2–179)	16 (1–441)
WHO classification						
AML with t(1;22)			3		3	0
AML with t(8;21)						6
AML with inv(16)				1	1	9
AML with t(15;17)						5
AML with t(9;11)						5
AML with inv(3)						1
AML with mutated NPM1				1	1	27
AML with biallelic CEBPA mutations						5
AML myelodysplasia-related changes				6	6	5
AML therapy-related						2
AML NOS, minimaldifferentiation						4
AML NOS, without maturation						8
AML NOS, with maturation						8
AML NOS, myelomonocytic						13
AML NOS, monoblastic/monocytic						12
AML NOS, erytroid						4
Not further classified				2	2	0

**Table 2:** Expression of markers in AMKL and non-AMKL patients ^a^.

	AMKL		Non-AMKL			Odds Ratio	95% CI	*p*
CD42a.CD61	66/72	92%	11/114	10%	*^#^	103.00	36.34 to 291.89	<0.0001
CD36	65/71	92%	68/114	60%	*^#^	7.32	2.93 to 18.32	<0.0001
CD71	65/72	90%	65/114	57%	*^#^	7.00	2.95 to 16.60	<0.0001
CD42b	52/64	81%	4/114	4%	*^#^	119.17	36.67 to 387.31	<0.0001
CD38	56/70	80%	97/114	85%		0.70	0.32 to 1.53	0.3722
CD33	56/71	79%	101/114	89%	^#^	0.48	0.21 to 1.08	0.0766
CD41	46/64	72%	15/114	13%	*^#^	15.18	7.13 to 32.31	<0.0001
CD7	50/71	70%	36/114	32%	*^#^	5.16	2.71 to 9.83	<0.0001
CD123	42/71	59%	88/114	77%	*^#^	0.43	0.22 to 0.82	0.0098
CD11b	27/70	39%	76/114	67%	*^#^	0.31	0.17 to 0.58	0.0002
HLADR	28/72	39%	87/114	76%	*^#^	0.20	0.10 to 0.37	<0.0001
CD15	25/70	36%	77/114	68%	*^#^	0.27	0.14 to 0.50	<0.0001
CD13	16/70	23%	80/114	70%	*^#^	0.20	0.11 to 0.38	<0.0001
CD64	9/70	13%	46/114	40%	*^#^	0.22	0.10 to 0.48	0.0002
CD14	6/70	9%	27/114	25%	*^#^	0.30	0.12 to 0.77	0.0127
CD105	6/71	8%	5/114	4%		2.01	0.59 to 6.86	0.2636
CD203c	2/72	3%	9/114	8%	^#^	0.33	0.07 to 1.59	0.1680
CD300e	2/70	3%	10/114	9%	^#^	0.31	0.07 to 1.44	0.1338
NG2	1/70	1%	18/114	16%	*^#^	0.08	0.01 to 0.59	0.0138

^a^ Data show the number and percentage of patients positive for a particular marker. A marker was considered to be expressed (i.e., to be positive) if the mean fluorescence intensity exceeded the value of 1000. * Significant difference in frequency between AMKL and non-AMKL (*p* < 0.05 by Fisher’s exact test); ^#^ Significant difference in mean fluorescence intensity between AMKL and non-AMKL (*p* < 0.05 by Mann–Whitney test).

## Data Availability

For original data, please contact the corresponding author.

## Supplementary Materials

The following supporting information can be downloaded at: https://www.mdpi.com/article/10.3390/cancers14061583/s1, Figure S1: Age and WBC count of AMKL and non-AMKL patients; Figure S2: Immunophenotypic profile of AMKL and non-AMKL patients; Figure S3: Expression of megakaryocytic markers in AMKL and non-AMKL patients; Figure S4: Immunophenotypic profile of three AMKL patients lacking expression of megakaryocytic markers; Figure S5: Multivariate analysis of CD34+/CD117+ cells from AMKL and non-AMKL patients; Figure S6: Expression of markers on maturation subsets of AMKL; Figure S7: Multidimensional analysis of maturation subsets of AMKL; Table S1: Detailed information from the 22 non-AMKL patients that expressed at least one megakaryocytic marker.

## References

[1] Arber D.A., Baumann I., Niemeyer C., Brunning R.D., Porwit A. (2017). Myeloid proliferations associated with Down Syndrome. WHO Classification of Tumours Iof Haematopoietic and Lymphoid Tissue.

[2] Arber D.A., Brunning R.D., Le Beau M.M., Falini B., Vardiman J.W., Porwit A., Thiele J., Foucar K., Dohner H., Bloomfield C.D. (2017). Acute myeloid leukemia with recurrent genetic abnormalities. WHO Classification of Tumours Iof Haematopoietic and Lymphoid Tissue.

[3] Arber D.A., Brunning R.D., Orazi A., Bain B.J., Porwit A., Le Beau M.M., Greenberg P. (2017). Acute myeloid leukemia with myelodysplasia-related changes. WHO Classification of Tumours Iof Haematopoietic and Lymphoid Tissue.

[4] Arber D.A., Brunning R.D., Orazi A., Porwit A., Peterson L.C., Thiele J., Le Beau M.M., Hasserjian R.P. (2017). Acute myeloid leukemia, NOS. WHO Classification of Tumours Iof Haematopoietic and Lymphoid Tissue.

[5] Oki Y., Kantarjian H.M., Zhou X., Cortes J., Faderl S., Verstovsek S., O’Brien S., Koller C., Beran M., Bekele B.N. (2006). Adult acute megakaryocytic leukemia: An analysis of 37 patients treated at M.D. Anderson Cancer Center. Blood.

[6] de Rooij J.D., Branstetter C., Ma J., Li Y., Walsh M.P., Cheng J., Obulkasim A., Dang J., Easton J., Verboon L.J. (2017). Pediatric non-Down syndrome acute megakaryoblastic leukemia is characterized by distinct genomic subsets with varying outcomes. Nat. Genet..

[7] Zhao G., Wu W., Wang X., Gu J. (2018). Clinical diagnosis of adult patients with acute megakaryocytic leukemia. Oncol. Lett..

[8] Hahn A.W., Li B., Prouet P., Giri S., Pathak R., Martin M.G. (2016). Acute megakaryocytic leukemia: What have we learned. Blood Rev..

[9] Hitzler J.K., Zipursky A. (2005). Origins of leukaemia in children with Down syndrome. Nat. Rev. Cancer.

[10] Gamis A.S., Smith F.O. (2012). Transient myeloproliferative disorder in children with Down syndrome: Clarity to this enigmatic disorder. Br. J. Haematol..

[11] Bernstein J., Dastugue N., Haas O.A., Harbott J., Heerema N.A., Huret J.L., Landman-Parker J., LeBeau M.M., Leonard C., Mann G. (2000). Nineteen cases of the t(1;22)(p13;q13) acute megakaryblastic leukaemia of infants/children and a review of 39 cases: Report from a t(1;22) study group. Leukemia.

[12] McElwaine S., Mulligan C., Groet J., Spinelli M., Rinaldi A., Denyer G., Mensah A., Cavani S., Baldo C., Dagna-Bricarelli F. (2004). Microarray transcript profiling distinguishes the transient from the acute type of megakaryoblastic leukaemia (M7) in Down’s syndrome, revealing PRAME as a specific discriminating marker. Br. J. Haematol..

[13] Yamato G., Park M.J., Sotomatsu M., Kaburagi T., Maruyama K., Kobayashi T., Nishi A., Sameshima K., Ohki K., Hayashi Y. (2021). Clinical features of 35 Down syndrome patients with transient abnormal myelopoiesis at a single institution. Int. J. Hematol..

[14] Athale U.H., Razzouk B.I., Raimondi S.C., Tong X., Behm F.G., Head D.R., Srivastava D.K., Rubnitz J.E., Bowman L., Pui C.H. (2001). Biology and outcome of childhood acute megakaryoblastic leukemia: A single institution’s experience. Blood.

[15] Helleberg C., Knudsen H., Hansen P.B., Nikolajsen K., Kjaersgaard E., Ralfkiaer E., Johnsen H.E. (1997). CD34+ megakaryoblastic leukaemic cells are CD38-, but CD61+ and glycophorin A+: Improved criteria for diagnosis of AML-M7?. Leukemia.

[16] Klairmont M.M., Hoskoppal D., Yadak N., Choi J.K. (2018). The Comparative Sensitivity of Immunohistochemical Markers of Megakaryocytic Differentiation in Acute Megakaryoblastic Leukemia. Am. J. Clin. Pathol..

[17] Langebrake C., Creutzig U., Reinhardt D. (2005). Immunophenotype of Down syndrome acute myeloid leukemia and transient myeloproliferative disease differs significantly from other diseases with morphologically identical or similar blasts. Klin. Padiatr..

[18] Lorsbach R.B. (2004). Megakaryoblastic disorders in children. Am. J. Clin. Pathol..

[19] van Dongen J.J., Lhermitte L., Bottcher S., Almeida J., van der Velden V.H., Flores-Montero J., Rawstron A., Asnafi V., Lecrevisse Q., Lucio P. (2012). EuroFlow antibody panels for standardized n-dimensional flow cytometric immunophenotyping of normal, reactive and malignant leukocytes. Leukemia.

[20] Kalina T., Flores-Montero J., Lecrevisse Q., Pedreira C.E., van der Velden V.H., Novakova M., Mejstrikova E., Hrusak O., Bottcher S., Karsch D. (2015). Quality assessment program for EuroFlow protocols: Summary results of four-year (2010–2013) quality assurance rounds. Cytom. A.

[21] Kalina T., Flores-Montero J., van der Velden V.H., Martin-Ayuso M., Bottcher S., Ritgen M., Almeida J., Lhermitte L., Asnafi V., Mendonca A. (2012). EuroFlow standardization of flow cytometer instrument settings and immunophenotyping protocols. Leukemia.

[22] Glier H., Novakova M., Te Marvelde J., Bijkerk A., Morf D., Thurner D., Rejlova K., Lange S., Finke J., van der Sluijs-Gelling A. (2019). Comments on EuroFlow standard operating procedures for instrument setup and compensation for BD FACS Canto, II., Navios and BD FACS Lyric instruments. J. Immunol. Methods.

[23] Bras A.E., Beishuizen A., Langerak A.W., Jongen-Lavrencic M., Te Marvelde J.G., van den Heuvel-Eibrink M.M., Zwaan C.M., van Dongen J.J.M., van der Velden V.H.J. (2018). CD38 expression in paediatric leukaemia and lymphoma: Implications for antibody targeted therapy. Br. J. Haematol..

[24] Bras A.E., de Haas V., van Stigt A., Jongen-Lavrencic M., Beverloo H.B., Te Marvelde J.G., Zwaan C.M., van Dongen J.J.M., Leusen J.H.W., van der Velden V.H.J. (2019). CD123 expression levels in 846 acute leukemia patients based on standardized immunophenotyping. Cytom. B Clin. Cytom..

[25] Bras A.E., Osmani Z., de Haas V., Jongen-Lavrencic M., Te Marvelde J.G., Zwaan C.M., Mejstrikova E., Fernandez P., Szczepanski T., Orfao A. (2021). Standardised immunophenotypic analysis of myeloperoxidase in acute leukaemia. Br. J. Haematol..

[26] Bottcher S., Engelmann R., Grigore G., Fernandez P.C., Caetano J., Flores-Montero J., van der Velden V.H.J., Novakova M., Philippe J., Ritgen M. (2021). Expert-independent classification of mature B-cell neoplasms using standardized flow cytometry: A multicentric study. Blood Adv..

[27] Yang W., Wang K., Zuo W. (2012). Neighborhood Component Feature Selection for High-Dimensional Data. J. Comput..

[28] Salakhutdinov R., Hinton G. Learning a Nonlinear Embedding by Preserving Class Neighbourhood Structure. Proceedings of the Eleventh International Conference on Artificial Intelligence and Statistics.

[29] Della Porta M.G., Travaglino E., Boveri E., Ponzoni M., Malcovati L., Papaemmanuil E., Rigolin G.M., Pascutto C., Croci G., Gianelli U. (2015). Minimal morphological criteria for defining bone marrow dysplasia: A basis for clinical implementation of WHO classification of myelodysplastic syndromes. Leukemia.

[30] Matsuo S., Nishinaka-Arai Y., Kazuki Y., Oshimura M., Nakahata T., Niwa A., Saito M.K. (2021). Pluripotent stem cell model of early hematopoiesis in Down syndrome reveals quantitative effects of short-form GATA1 protein on lineage specification. PLoS ONE.

[31] Boztug H., Schumich A., Potschger U., Muhlegger N., Kolenova A., Reinhardt K., Dworzak M. (2013). Blast cell deficiency of CD11a as a marker of acute megakaryoblastic leukemia and transient myeloproliferative disease in children with and without Down syndrome. Cytom. B Clin. Cytom..

[32] Savasan S., Buck S., Raimondi S.C., Becton D.L., Weinstein H., Chang M., Ravindranath Y. (2006). CD36 (thrombospondin receptor) expression in childhood acute megakaryoblastic leukemia: In vitro drug sensitivity and outcome. Leuk Lymphoma.

