# Supplementary material for: Immunophenotypic Analysis of Acute Megakaryoblastic Leukemia: A EuroFlow Study
Source: Cancers (Basel). 2022 Mar 21;14(6):1583. doi: 10.3390/cancers14061583 (PMC8946548; doi:10.3390/cancers14061583)
Supplement: Supplementary file 1 [file cancers-14-01583-s001.zip › cancers-1613040-supplementary.pdf]

A. B.

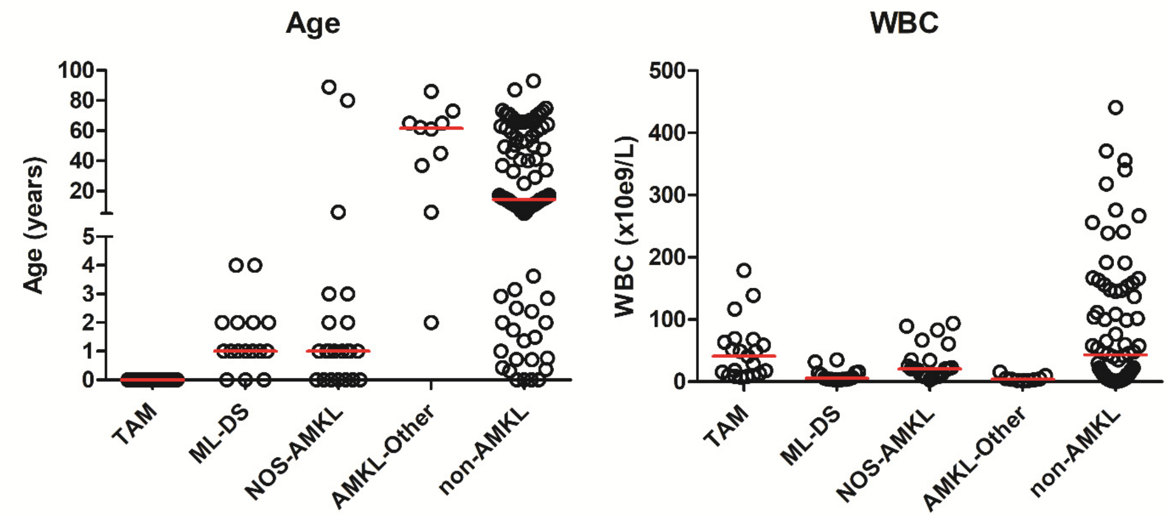

Supplementary Figure S1. A. Age (in years) of the patients included in the various subgroups. Age was significantly different between the all groups, except for ML-DS versus NOS-AMKL (Kruskal-Wallis test, if p<0.05 followed by Mann-Whitney test, p<0.05). **B.** WBC counts (10e9/L) of the patients included in the various subgroups. WBC counts were significantly lower in ML-DS and AMKL-Other as compared to the other subgroups (Kruskal-Wallis test, if p<0.05 followed by Mann Whitney test, p<0.05). Subgroups included TAM (n=24), ML-DS (n=16), NOS-AMKL (n=22), AMKL-Other (n=10), and non-AMKL (n=114). Red bars indicate median values.

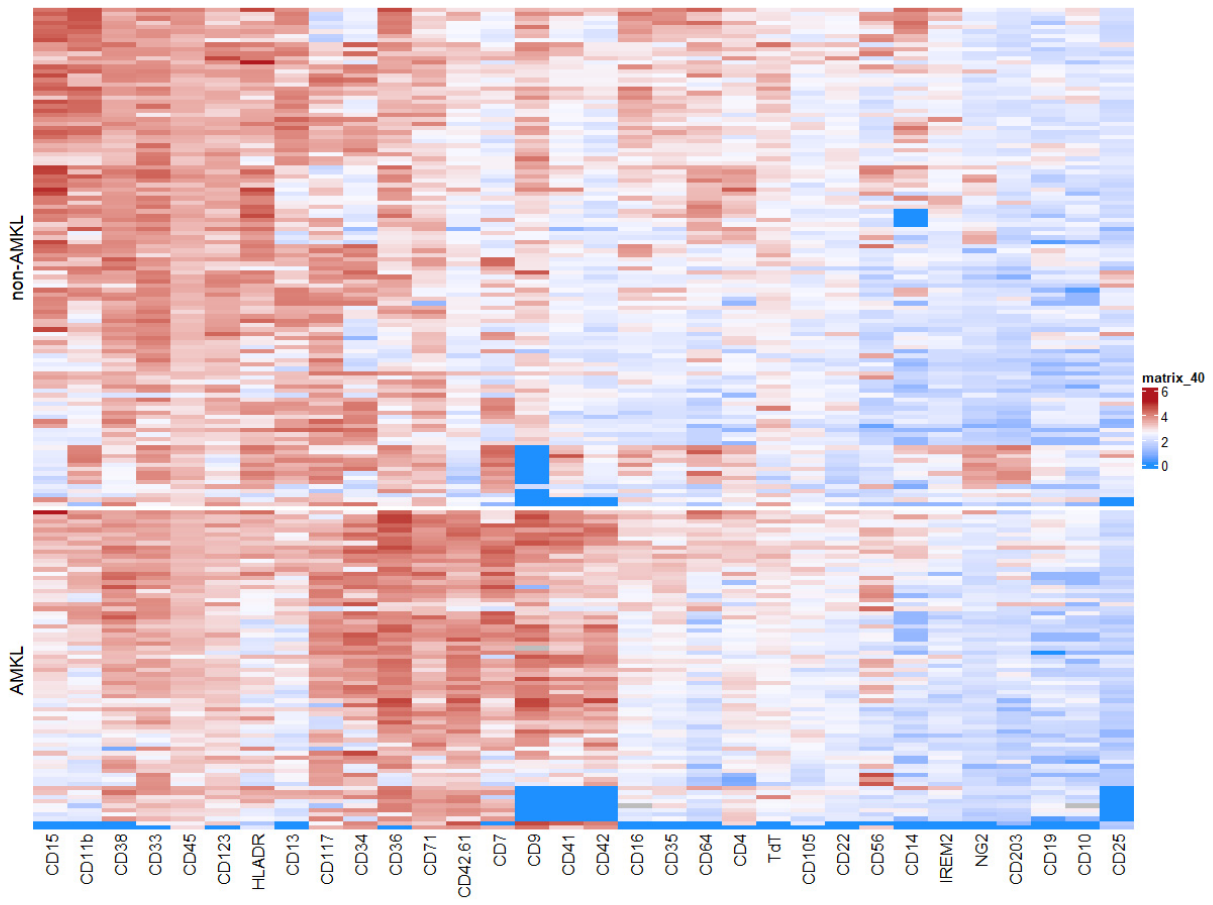

Supplementary Figure S2. Immunophenotypic profile of AMKL and non-AMKL patients. Data show the mean fluorescence intensity of the markers as specified on the x-axis, whereas each row represents a specific AML patient. Data were log10 transformed. Missing data are represented as 0 (darkest blue color).

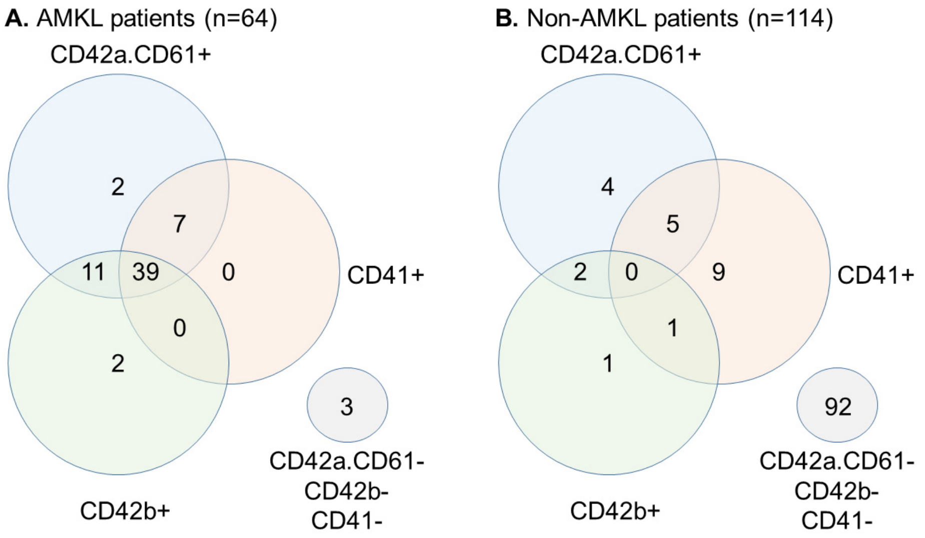

Supplementary Figure S3. Expression of megakaryocytic markers in AMKL (**A.**) and non-AMKL (**B.**) patients. Only AMKL and non-AMKL patients with complete flowcytometric data from EuroFlow AML panel tube 6 and 7 available were included.

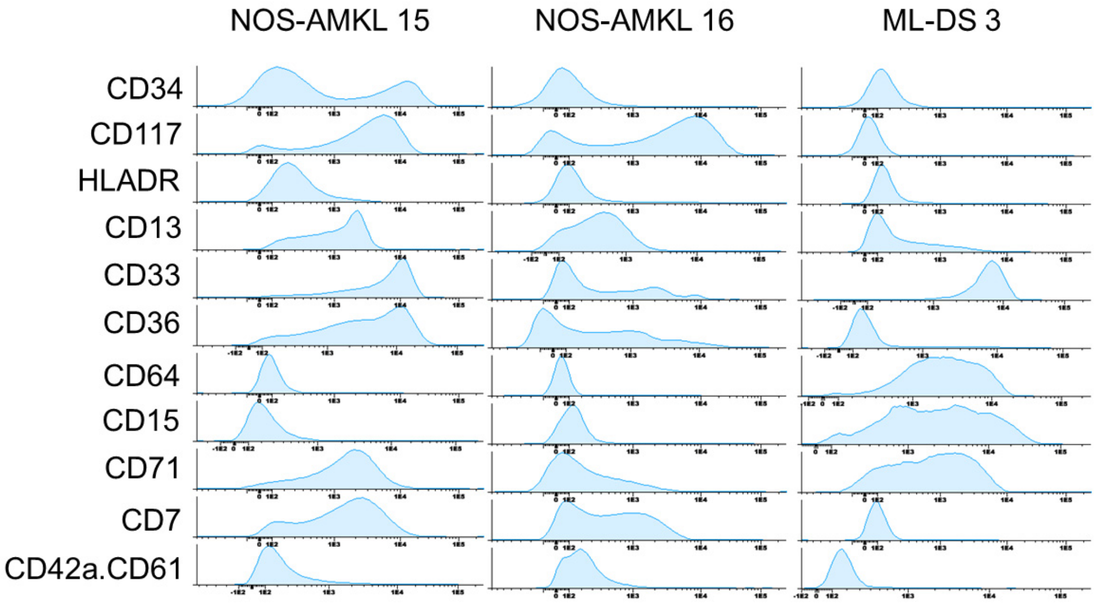

Supplementary Figure S4. Immunophenotypic profile of three AMKL patients lacking expression of CD41, CD42a, CD42b and CD61. Histograms for various markers are shown, with MFI values on the x-axis and number of events on the y-axis.

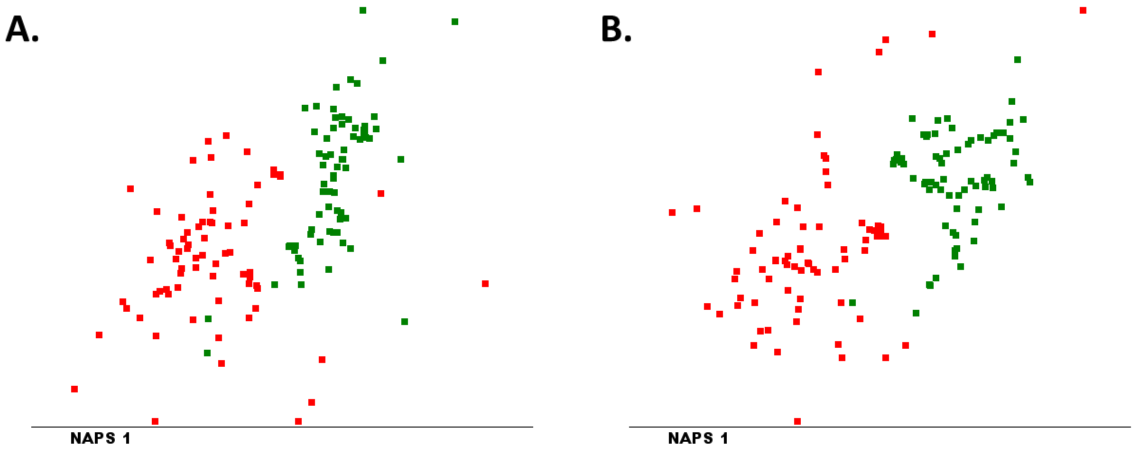

Marker	Contribution
CD42a.CD61	62%
CD42b	37%
CD36	1%

Marker	Contribution
CD42a.CD61	99%
CD36	1%

Supplementary Figure S5. Multivariate analysis of CD34+/CD117+ cells from non-AMKL (green symbols) and AMKL patients (red symbols) using EuroFlow AML tube 1-7 (**A.**) or tube 1-6 (**B.**). Pattern classification was performed using NAPS and the markers contributing to the pattern classification are shown in the bottom part of the figure. Only cases with >100 CD34+/CD117+ cells were included in the analysis (72 AMKL, 71 non-AMKL).

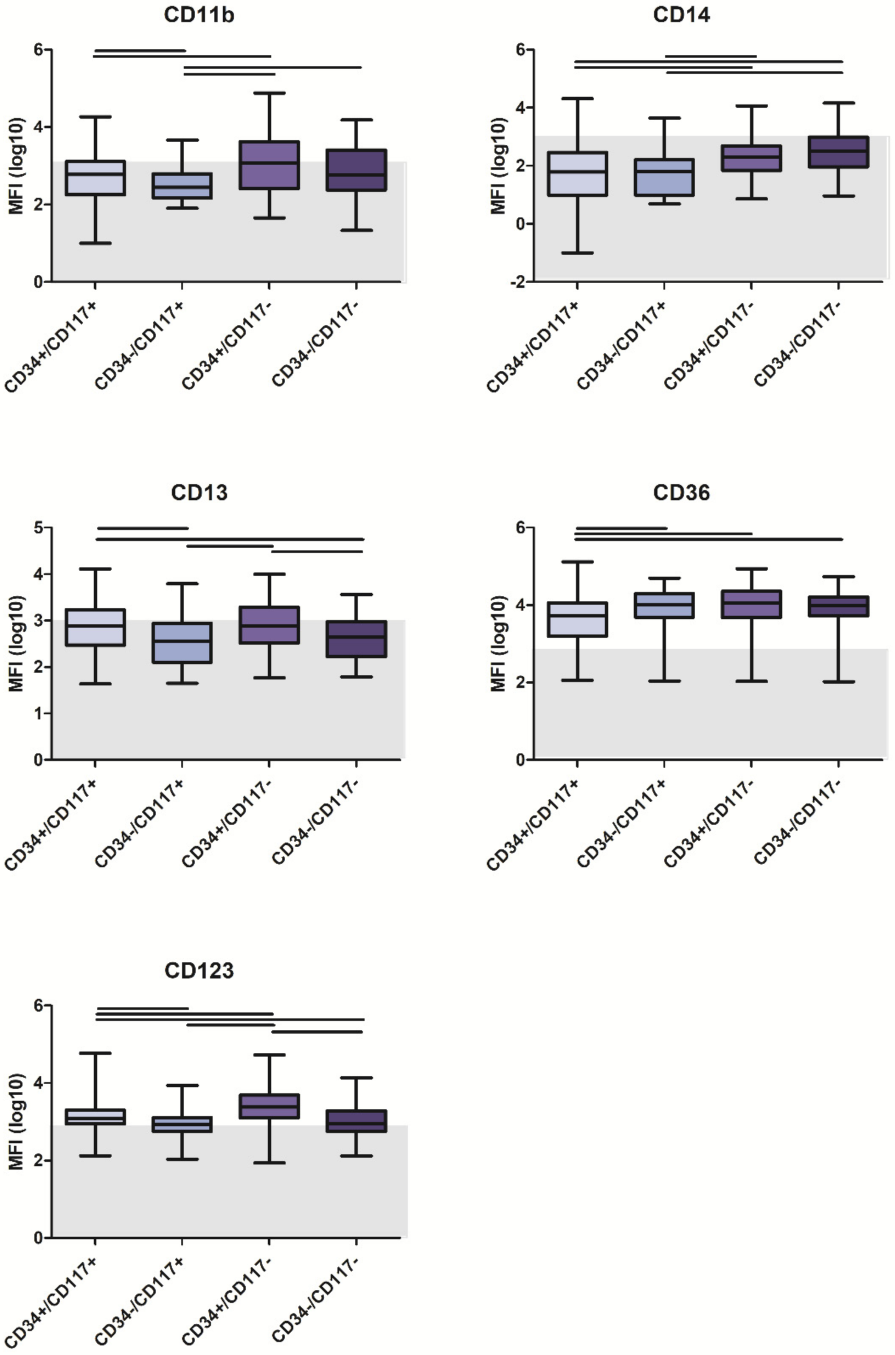

Supplementary Figure S6. Expression of markers on AMKL maturation subsets CD34+/CD117+, CD34-/CD117+, CD34+/CD117-, and CD34-/CD117-. Data represent the MFI values after log10 transformation. Horizontal base between populations indicate significant differences (Kruskal-Wallis, if *p*<0.05 followed by

Mann Whitney test; *p*<0.05). The grey zone indicates MFI levels <1000, markers with such MFI values were considered to be negative.

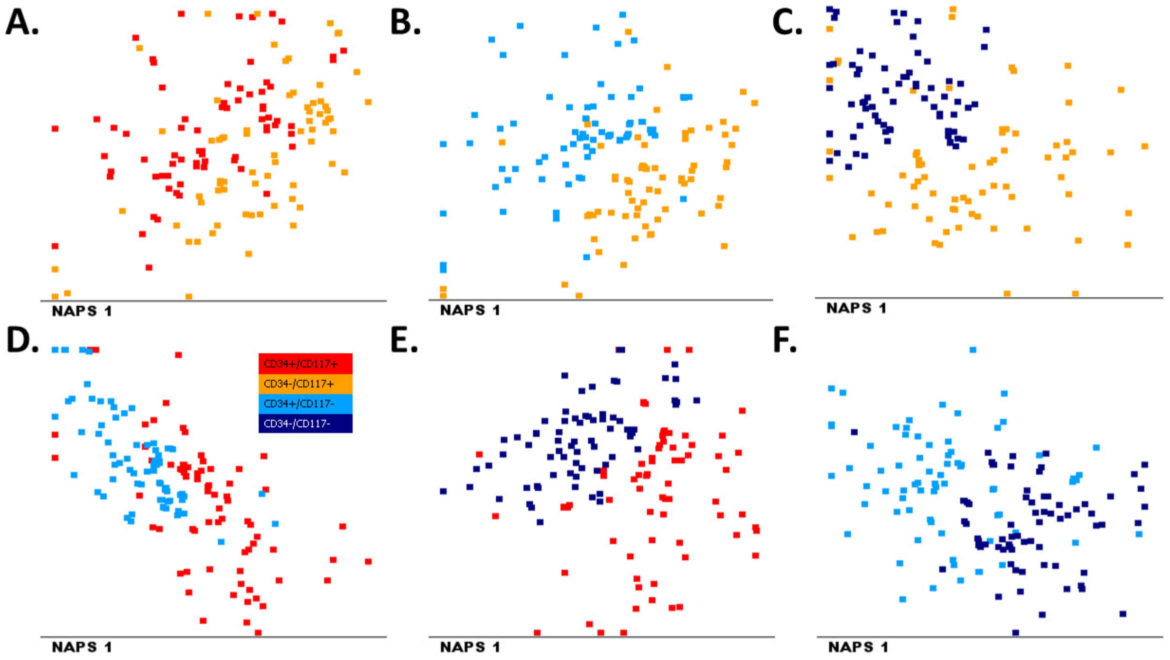

Supplementary Figure S7. Multidimensional analysis of four maturation stages of AMKL cells. **A.** CD34+/CD117+ versus CD34-/CD117+ (contributing markers: CD10 (14%); HLADR (9%), CD33 (8%)); **B.** CD34+/CD117+ versus CD34+/CD117- (contributing markers: CD10 (44%); CD22 (40%), CD14 (15%)); **C.** CD34+/CD117+ versus CD34-/CD117- (contributing markers: CD14 (96%); CD7 (2%), CD15 (1%)); **D.** CD34-/CD117+ versus CD34+/CD117- (contributing markers: CD14 (99%); HLADR (0.1%), CD13 (0.1%)); **E.** CD34-/CD117+ versus CD34-/CD117- (contributing markers: CD14 (57%); CD38 (25%), CD33 (15%)); **F.** CD34+/CD117- versus CD34-/CD117- (contributing markers: CD16 (33%), CD35 (30%), CD36 (12%)). Note that markers CD34 and CD117 were excluded from the analysis, as they were were used for subgroup definitions.

Supplementary Table S1. Detailed information from the 22 non-AMKL patients that expressed at least one megakaryocytic marker. Markers with an MFI value above 1000 are marked in color.

Patient	CD117	CD13	CD11b	HLADR	CD14	CD64	CD36	CD33	CD42a.CD	CD42	CD41	final diagnosis (WHO)	gender	age
1	869	8396	16006	2778	2907	3076	6050	5862	1418	493	2092	Therapy-related myeloid neoplasms	M	6
2	3127	6450	638	229	160	345	471	12753	423	1197	460	AML with t(15;17)	M	17
3	12055	1602	1696	1499	54	976	6245	1820	1882	1255	842	Acute myelomonocytic leukemia	F	14
4	4025	8257	15739	1354	196	4121	1176	7712	1090	499	818	Acute myelomonocytic leukemia	F	9
5	874	4449	9974	10215	532	4117	7801	3153	2878	480	4749	Acute monoblastic and monocytic leukemia	F	16
6	633	5303	2620	110775	93	2228	3089	5939	1050	227	516	Acute myelomonocytic leukemia	F	10
7	938	10405	8766	447	520	947	1120	1963	549	822	1212	AML with t(15;17)	M	10
8	51	8060	33233	668	7527	1308	9410	11654	1967	304	489	AML with t(9;11)	M	0
9	1416	11991	1225	9809	437	1176	1304	6200	1145	574	2028	AML with t(9;11)	F	15
10	64	10229	34056	929	6545	1594	12414	4013	3315	879	4672	AML with t(9;11)	F	0
11	1697	338	3888	51319	355	1866	7863	7055	3100	396	7493	AML with t(9;11)	M	0
12	54	702	9406	4225	7993	3400	16248	4879	846	515	1750	AML with t(9;11)	F	10
13	3160	3917	10198	2014	272	2245	3663	6529	135	983	32823	AML with myelodysplasia-related changes	M	62
14	385	2014	9008	8727	1262	26207	5940	3783	141	252	2050	AML with mutated NPM1	F	55
15	502	5251	8467	13354	1800	8540	9384	11022	248	362	1507	AML with mutated NPM1	M	71
16	2921	2273	4462	8062	241	2279	2545	842	75	208	1108	AML with mutated CEBPA	F	40
17	5469	5630	4312	11354	379	16700	3234	7884	60	217	1662	AML with mutated NPM1	M	29
18	8337	8896	11885	3087	1274	2510	2827	5404	102	319	2505	AML with maturation	F	63
19	774	10915	18047	1417	211	600	2570	15901	1140	682	333	AML with mutated NPM1	M	50
20	369	1913	6207	10453	1182	158	3917	1188	1627	1165	464	AML without maturation	F	69
21	2285	5629	30661	3902	939	372	1496	10429	621	586	1763	Acute myelomonocytic leukemia	F	73
22	883	5971	37417	45657	16365	22542	42191	14038	611	1656	1215	AML with mutated NPM1	F	65
